# Understanding the Role of ATP Release through Connexins Hemichannels during Neurulation

**DOI:** 10.3390/ijms24032159

**Published:** 2023-01-21

**Authors:** Lina Mariana Tovar, Carlos Felipe Burgos, Gonzalo E. Yévenes, Gustavo Moraga-Cid, Jorge Fuentealba, Claudio Coddou, Luisa Bascunan-Godoy, Claudio Catrupay, Angel Torres, Patricio A. Castro

**Affiliations:** 1 Laboratory of Physiology and Pharmacology for Neural Development, LAND, Departamento de Fisiología, Facultad de Ciencias Biológicas, Universidad de Concepción, Concepción 4030000, Chile; 2 Departamento de Fisiología, Facultad de Ciencias Biológicas, Universidad de Concepción, Concepción 4030000, Chile; 3 Departamento de Ciencias Biomédicas, Facultad de Medicina, Universidad Católica del Norte, Coquimbo 1781421, Chile; 4 Departamento de Botánica, Facultad de Ciencias Naturales y Oceanográficas, Universidad de Concepción, Concepción 4030000, Chile

**Keywords:** neural tube defects, neurulation, purinergic signalling, connexins hemichannels, ATP

## Abstract

Neurulation is a crucial process in the formation of the central nervous system (CNS), which begins with the folding and fusion of the neural plate, leading to the generation of the neural tube and subsequent development of the brain and spinal cord. Environmental and genetic factors that interfere with the neurulation process promote neural tube defects (NTDs). Connexins (Cxs) are transmembrane proteins that form gap junctions (GJs) and hemichannels (HCs) in vertebrates, allowing cell-cell (GJ) or paracrine (HCs) communication through the release of ATP, glutamate, and NAD^+^; regulating processes such as cell migration and synaptic transmission. Changes in the state of phosphorylation and/or the intracellular redox potential activate the opening of HCs in different cell types. Cxs such as Cx43 and Cx32 have been associated with proliferation and migration at different stages of CNS development. Here, using molecular and cellular biology techniques (permeability), we demonstrate the expression and functionality of HCs-Cxs, including Cx46 and Cx32, which are associated with the release of ATP during the neurulation process in *Xenopus laevis*. Furthermore, applications of FGF2 and/or changes in intracellular redox potentials (DTT), well known HCs-Cxs modulators, transiently regulated the ATP release in our model. Importantly, the blockade of HCs-Cxs by carbenoxolone (CBX) and enoxolone (ENX) reduced ATP release with a concomitant formation of NTDs. We propose two possible and highly conserved binding sites (N and E) in Cx46 that may mediate the pharmacological effect of CBX and ENX on the formation of NTDs. In summary, our results highlight the importance of ATP release mediated by HCs-Cxs during neurulation.

## 1. Introduction

The formation of the central nervous system (CNS) occurs relatively early during embryonic development in a process known as neurulation, which is a morphogenetic complex process that requires the coordination of cellular and molecular events, leading to the formation of the neural tube [1]. This process is highly conserved between vertebrates such as amphibians and humans, taking place between 14.5–21.5 h post fertilization (hpf, stages 12.5–20) in *Xenopus laevis* (www.xenbase.org/, RRID:SCR_003280, accessed on 1 January 1999) [2] and between days 20–28 in humans (Carnegie Stages, CS 9–13, [3]). Signaling pathways activated by WNT (Wingless-int), FGFs (Fibroblast Growth Factors), and BMPs (Bone Morphogenetic Proteins) actively participate in this process [4,5,6]. These, and other molecules, regulate cellular events such as planar cell polarity and convergent extension [6], necessary for the proper closure of this structure, which generates the brain and spinal cord [7,8]. The incomplete closure of the neural tube results in neural tube defects (NTDs) [9,10]. NTDs are the most common CNS birth defect worldwide, with a prevalence of 1 per 1000 established pregnancies [7,11]. The causes of NTDs are multiple, being both genetic and environmental [12,13,14,15]. 

In addition to WNT, BMP, and FGFs signaling, neurotransmitters (NTs) participate in early events during development, such as the proliferation of neural precursors [16], differentiation and migration [17,18,19,20]. Other processes, such as oriented migration and proliferation of precursors from the neural plate [21] and the closure of the neural tube [22], have been associated as well. These antecedents reveal that NTs are relevant actors in development, however, their specific participation in neurulation has not been deciphered.

ATP is an NT and neuromodulator [20,23,24] associated with the synthesis and release of trophic factors such as fibroblast growth factor 2 (FGF2), nerve growth factor (NGF), and neurotrophin 3 (NT-3) [20,25,26]. Then, the purinergic signaling increases [Ca^2+^]i modulating the propagation of calcium waves, which play an important role in embryonic development and neural tube formation [27]. The P2X(2-7) and P2Y(1,8) receptors and the ectonucleoside triphosphate diphosphohydrolase-2 (eNTPDase-2) enzyme, modulate ATP signaling in neurulation, early embryonic neurogenesis, and brain development in rats [25,28,29,30]. Complementarily, ATP-mediated signaling before synaptogenesis has been proposed [21,25,28,29].

NTs are classically released through synaptic vesicles [31,32,33], however, an alternative liberation mechanism has been described with the participation of connexins [34,35,36,37,38,39]. Vesicular release during neurulation is uncertain, thus it will be important to elucidate the participation of the connexin-mediated release of NTs during neurulation.

Connexins (Cxs) allow direct intercellular communication between cytoplasms as gap junctions, assembled in each membrane as homo or heteromeric hemichannel (HC) formed by six subunits, also known as a connexon. The specific composition of the HC gives it specific characteristics such as selective permeability to different molecules [40,41,42]. Nearly 20 Cxs have been described to now, classified according to molecular weight (i.e., cx 46, 46 KDa) or similarity (alpha, beta) [43]. Different cell types express specific Cxs that can vary according to the stage of development. For instance, Cx43 and Cx46 are expressed in early migrating neural crest cells [44], while Cx26, Cx32, and Cx43 are expressed in early neurogenesis [45]. Cx26, Cx32, Cx43, and Cx45 are found in embryonic development in the ventricular zone (ZV) of the lateral ventricles [38,46], in the postnatal subventricular zone (SVZ) in late neurogenesis [47] and in embryonic and early postnatal neurogenesis and gliogenesis [48]. However, which Cxs are present during neurulation and their functional role(s), are still unknown.

In addition to direct cell-cell communication, HCs can release molecules such as ATP, glutamate, glutathione, prostaglandin E2, and NAD^+^, which function as autocrine/paracrine messengers regulating processes such as growth, apoptosis, and others [36,37,39,41,49,50,51].

The opening probability of HCs is low, but it increases through phosphorylation/dephosphorylation [51,52,53,54,55,56]; low external Ca^2+^ concentration [37] and redox potentials [49,51,57,58,59,60]. In addition, in the open state, HCs can mobilize exogenous fluorescent and low molecular weight (<1 kDa) molecules such as ethidium bromide (EtBr) and Lucifer yellow (LY) widely used to detect functional hemichannels [37,39,60,61,62,63]. Then, drugs such as carbenoxolone (CBX) and enoxolone (ENX) [64] can block the functionality of Cxs and modulate the uptake of these molecules, but it is unknown how these drugs would modulate the connexin action.

Here, we demonstrate the presence and functionality of HCs-Cxs (e.g., Cx46/Cx32) in ATP release during the neurulation process in Xenopus laevis, showing the relevant presence of Cx46, Cx32 transcripts while their pharmacological blockade (CBX and ENX) causes NTDs. In addition, their functionality as HCs is transiently regulated by FGF2 and/or intracellular redox potentials using Dithiothreitol (DTT), which can trigger the activation of purinergic signaling. Finally, in silico analysis showed two sites (N and E) of interaction between Cx46 and CBX/ENX, which are highly conserved among Cxs. Our results suggest that HCs-Cxs participate in the neurulation process triggering the release of ATP, and their pharmacological blockade causes NTDs.

## 2. Results

### 2.1. Transcript Transcript Profile of Cxs during Neurulation in Xenopus laevis 

**Figure 1:**
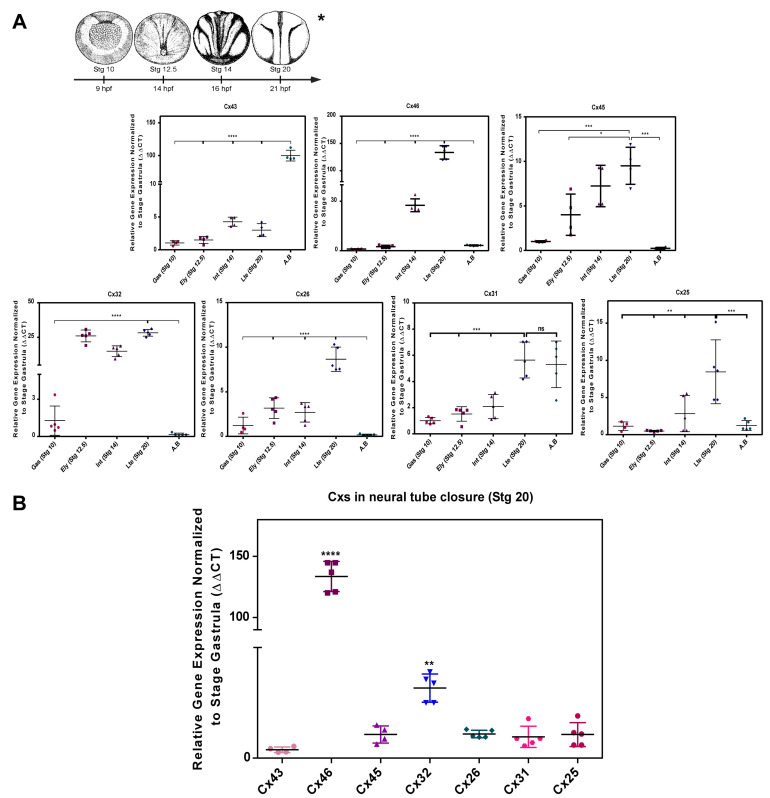
Cx46 and Cx32 transcripts are enriched during *Xenopus laevis* Neurulation. (**A**) RT-qPCR of Cx43, Cx46, Cx45, Cx32, Cx26, Cx31, and Cx25 in the process of formation of the neural tube of *Xenopus laevis* N = 4. (**B**) Comparative analysis of expression of Cxs in the closure of the neural tube of *Xenopus laevis* N = 4. Transcriptional expression was normalized to the sub-1 control gene. One-way ANOVA, Dunnett’s correction, (* = *p* < 0.05; ** = *p* < 0.01; *** = *p* < 0.001; **** = *p* < 0.0001; ns = not significant). The error bars of the data correspond to the standard deviation.

Conventional RT-PCR analysis showed the presence of Cx43, Cx46, Cx45, Cx32, Cx26, Cx31, and Cx25 mRNA in both the gastrula stage (Stg 10), early neurula (Stg 12.5), intermediate neurula (Stg 14), late neurula (Stg 20), and adult brain (AB) (Figure S1). Using RT-qPCR, we confirmed the positive mRNA amplification of Cx43, Cx46, Cx45, Cx32, Cx26, Cx31, and Cx25 in Stg 10, 12.5, 14, 20, and AB as shown in Figure 1A. It was evidenced that the mentioned Cxs tend to progressively increase their signal concerning the expression levels reported in the gastrula stage (Stg 10) (Figure 1A). The variations of Cx45, Cx32, Cx31, and Cx25 are not statistically significant, while Cx46 increased its expression 100-fold in Stg 20 (Figure 1A). An analysis of the relative mRNA levels showed that Cx46 would be 120 times more abundant than Cx43 and Cx45, while Cx32 was 30 times more abundant than Cx26, Cx31, and Cx 25 (Figure 1B), which would suggest the participation of these Cxs during neural tube formation Figure 1B. Additional experiments, show the presence of Cx46 protein during the different stages of neurulation in *Xenopus laevis* using western blot, establishing a positive association between Cx46 mRNA transcripts and the expression of the protein (Figure S4). Future experiments must be performed to confirm the presence of Cx32 and other Cxs. 

### 2.2. Pharmacological Blockade of Cxs in Neurulation Induces NTDs

**Figure 2:**
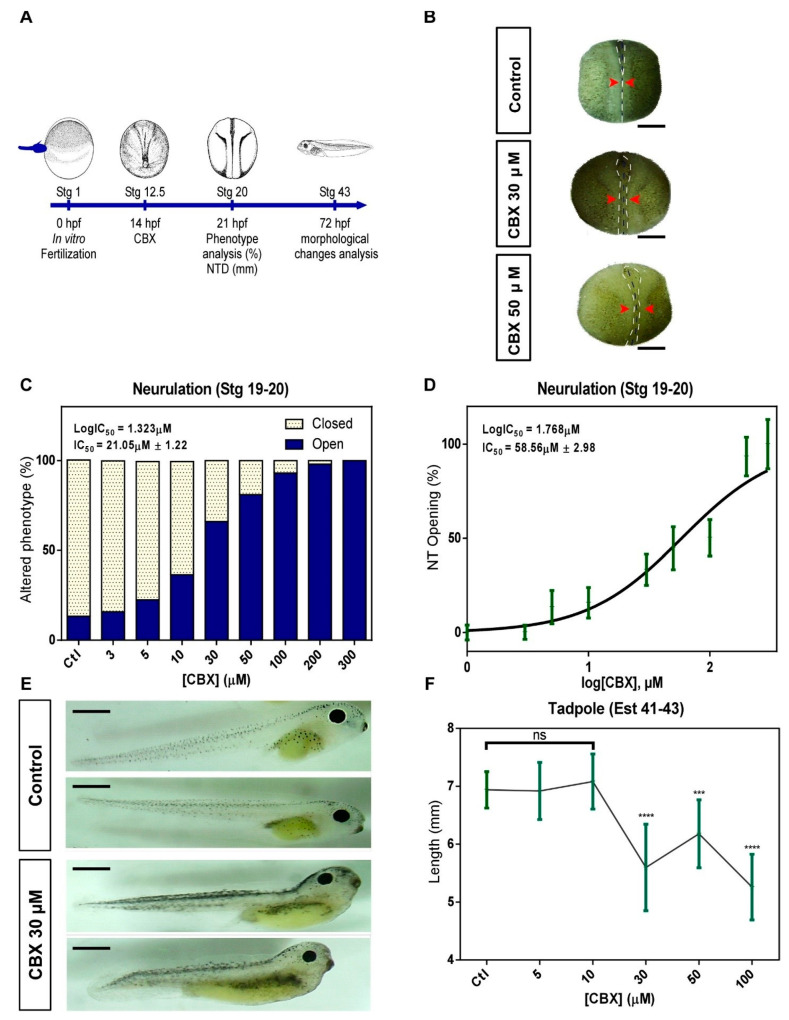
Pharmacological blockade of Cxs with CBX induces NTDs. (**A**) The diagram depicts the stage (Stg) where CBX was initially applied to embryos (Stg 12.5 = 14 hpf). (**B**) Representative photographs of embryos at stg 20 under control conditions (top) or treated with CBX (30 or 50 μM, middle and bottom, respectively). Dashed white lines mark the border between superficial neural and non-neural ectoderm, and dashed black lines indicate midline. Red arrowheads indicate the distance of neural folds (closed phenotype = normal; open phenotype = abnormal); scale bar = 500 μm. (**C**) The graph shows the percentage of open and closed phenotypes of embryos treated in the absence (control = ctl) or presence of CBX (3–300 µM; IC_50_ = 21.05 μM ± 1.22). (**D**) Quantification of open phenotype in embryos at stg 20 treated with CBX (IC_50_ = 58.56 μM ± 2.98). (**E**) The phenotype of tadpoles (stg 41–43) treated with CBX at stg 12.5–20. Scale bar = 1 mm. (**F**) The graph shows the effect of CBX on the length of *Xenopus* tadpoles. Independent fertilized embryos (N = 6) were analyzed for conditions (10 experimental replicates). One-way ANOVA, Dunnett’s correction, (*** = *p* < 0.001; **** = *p* < 0.0001; ns = not significant). Error bars represent the standard deviation.

To analyze the functional involvement of Cxs in neural tube formation, we used widely used blockers of Cxs [64], CBX (Figure 2), and ENX (Figure S2), to assess effects at early stages of embryonic development as neurulation (12.5–20) and tadpole (40–45) stages (Figure 2A and Figure S2A). The embryos were treated with increasing concentrations of CBX/ENX from Stg 12.5 until reaching the late neurula stage (Stg 20) (Figure 2A and Figure S2A), showing important morphological changes from 30 μM (CBX) (Figure 2B) and 50 μM (ENX) (Figure S2B). CBX and ENX caused a significant delay in tube formation vs. controls, showing an anterior opening of the neural tube in the embryo at 30 μM (CBX) (Figure 2B) and 30 μM (ENX) (Figure S2B). Concentration increment extends alteration to the entire anteroposterior axis (Figure 2B and Figure S2B). Phenotype quantification (open neural tube) showed that initially 20.2% (Figure 2C) and 15% (Figure S2C) of control embryos presented open phenotype incremented to 66.5% and 65.5% in embryos exposed to 30 μM CBX (Figure 2C) and 50 μM ENX (Figure S2C). At higher concentrations, values increase to 81.1% (Figure 2C) and 84.4% (Figure S2C), while at concentrations ≥ 100 μM, 100% of embryos present alterations (Figure 2C and Figure S2C). The concentration-response curve shows EC_50_ values for openings of 21.05 ± 1.22 μM (Figure 2C) and 47.83 ± 2.31 μM, CBX and ENX respectively (Figure S2C). Regarding open severity, while 30 μM CBX (Figure 2D) and 50 μM ENX (Figure S2D) caused 33.3% of neural tube opening (0.17 mm ± 0.05 and 0.14 ± 0.04 respectively), 58.56 ± 2.98 μM CBX (Figure 2D) and 66.74 ± 4.19 μM ENX (Figure S2D) generate a 50% of open vs. control embryos which presented a 2%. Finally, we evaluated the morphological changes (length in mm) induced by the transient exposure of Cxs blockers during neurulation in tadpoles (Stg 40–45). Embryos treated with 30 μM CBX presented altered phenotypes with curved anteroposterior axis (Figure 2E), evidencing alterations in their spinal cord formation. In 100 and 300 μM CBX, we observed growth inhibition and subsequent degeneration and/or death. The length of the embryos significantly varied from 6.93 ± 0.14 mm (control) to 5.59 ± 0.18mm (CBX) (Figure 2F), indicating that treatment with CBX generates alterations during neurulation which are later manifested in Stg 40–45. Embryos exposed to 30 μM ENX did not show altered phenotypes at the tadpole stage (Stg 40–45) (Figure S2E), but in the range of 100–300 μM, we showed complete deterioration of the embryos and subsequent death (Figure S2F). These data allow us to establish that CBX and ENX cause alterations in the neurulation process, but CBX generates extended effects in later stages of development (Tadpole Stage).

Considering that the more representative Cxs during neurulation will be 46 and 32 (Figure 1B) which form hemichannels (HCs) [65] and the use of CBX/ENX generate NTDs (Figure 2 and Figure S2), we decided to examine whether the HCs-Cxs activity participates in the phenotypic changes of neural tube closure (NTDs). This analysis was determined through the incorporation of the fluorescent dye Lucifer yellow (LY), which can be intracellularly incorporated from extracellular space by an HC-Cx mechanism [37,39,60,61,62,63,66].

### 2.3. Functional Analyses of HCs-Cxs In Vivo by Redox Potentials and FGF2 Using Lucifer Yellow Permeability Assay

**Figure 3:**
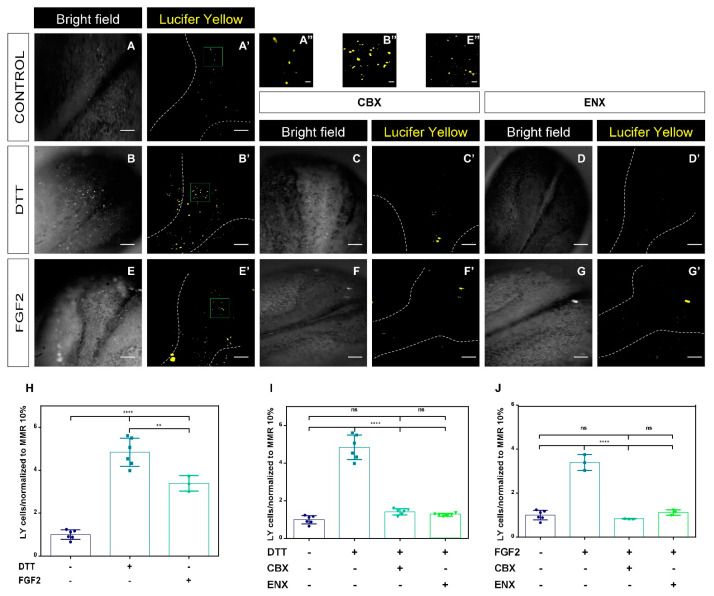
Lucifer Yellow uptake is modulated by FGF2 and DTT in neural plates. (**A**–**G**) Representative images of the neural tube closure process in the control condition, DTT, DTT/CBX, DTT/ENX, FGF2, FGF2/CBX, and FGF2/ENX. (**A’**–**G’**) Neural plate cell fluorescence, illustrating LY uptake in control conditions, DTT, DTT/CBX, DTT/ENX, FGF2, FGF2/CBX, and FGF2/ENX. Scale bar: 200 µm. The upper panel images show a magnification of the boxes in (**A’**,**B’**,**E’**) signaling LY uptake, respectively. Scale bar (**A’’**,**B’’**,**E’’**): 20 μm. Scale bar magnified boxes: 20 μm. (**H**) Comparison of positive LY cells under redox potential (DTT) and FGF2 conditions, quantification normalized to control condition (MMR 10%). (**I**) Quantification of LY cells after exposure to DTT. (**J**) Quantification of LY cells after exposure to FGF2. N = 3 groups of independent fertilized embryos and 5 replicates for each condition. One-way ANOVA, Bonferroní comparison test, (** = *p* < 0.01; **** = *p* < 0.0001; ns = not significant). Error bars for data represent standard deviation.

Embryos at stage 12.5 were treated with 20 ng/mL of FGF2, 1 mM of DTT, 30 μM of CBX, and 50 μM of ENX for 7 h [55,59]. LY uptake was determined through changes in the incorporation of LY fluorescence of neural plate cells from embryos at Stg 20. LY is unable to cross the plasma membrane itself and it is only incorporated in cells through HC-Cxs and/or Pnxs. Neural plate cells in the control condition (Figure 3A,A’) showed a detectable permeability to LY, confirming the presence and functionality of HCs-Cxs, during neurulation. Embryos treated with DTT and FGF2 showed a significant increase in LY uptake (Figure 3B,B’,E,E’,H–J) compared to the control condition, an effect that was inhibited by CBX and ENX (Figure 3C’,D’,F’,G’).

Embryos treated with DTT and FGF2 showed a 3-6-fold increase in their uptake level, compared to the control condition (Figure 3H). In addition, we observed significant differences between the embryos exposed to DTT and FGF2, showing greater permeability under redox conditions (Figure 3H). Treatment with CBX and ENX significantly decreased the uptake of LY-like control levels (*p* < 0.05) (Figure 3C,I,J). Our data suggest that the LY permeability of the neural plate cells induced by treatment with DTT and FGF2 and consequent reduction with CBX/ENX could be dependent on HCs-Cxs activity.

### 2.4. Evaluation of HCs-Cxs Functionality via ATP Release Induced by Redox Potentials and FGFs 

In agreement with our previous results, complementarily, we decided to evaluate the functionality of HC-Cxs measuring the extracellular release of ATP, a mechanism that has been previously shown to be dependent on Cxs [36,37,67]. In cells expressing HCs-Cxs, the intracellular redox potential [49,57,59] and the FGFs signaling [53,54,55], induce the release of ATP, glutamate, and NAD^+^. 

**Figure 4:**
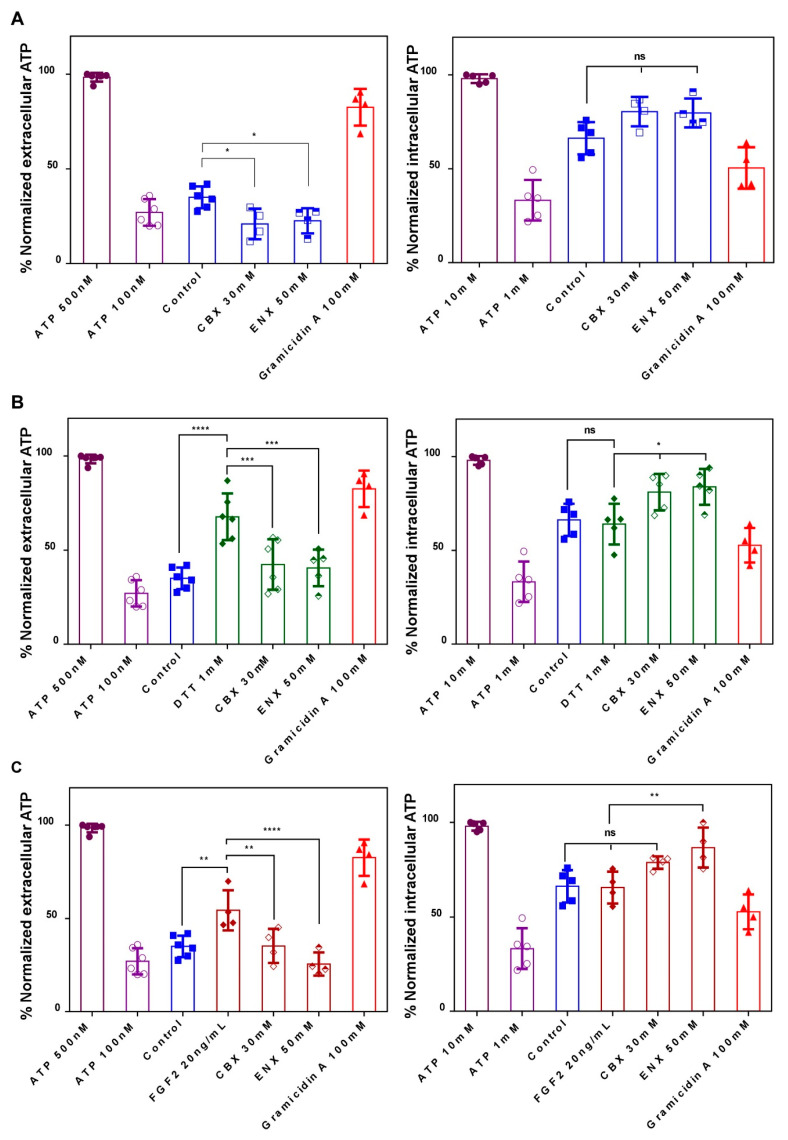
ATP Release during neurulation is regulated by HCs-Cxs modulators. Using luminescence assays (**A**), extracellular and intracellular ATP (nM) were quantified and normalized to the calibration curve. (**B**) Embryos treated with redox potentials. (**C**) Embryos treated with mitogenic factors. The results correspond to at least four experiments conducted independently (N = 4), for each condition. One-way ANOVA, Bonferroní comparison test, (* = *p* < 0.05; ** = *p* < 0.01; *** = *p* < 0.001; **** = *p* < 0.0001; ns = not significant.). The error bars for the data represent the standard deviation.

We decide to evaluate if DTT and FGF2 contribute to the ATP release promoting HCs-Cxs opening, similarly to LY assays, determining the ATP concentration in the extracellular and intracellular medium of embryos treated with CBX and ENX through luciferase assay (Figure 4). According to previous works [53,54], we established a dynamic range between 0.01–1 µM of ATP, which were normalized (%) and then used as a quantifiable representation of the relative intracellular and extracellular levels of ATP. Under basal conditions, released ATP was 37% of our scale (~0.180 μM), and reduced significantly to 20% and 23% with CBX and ENX, respectively (*p* < 0.05) (Figure 4A). Gramicidin A, which generates pores in the plasma membrane, allowed ATP and others to leak extracellularly, reaching a concentration of 82% (~0.4 μM). 

In the presence of DTT and FGFs, the released ATP significantly (*p* < 0.05) reached relative values of 68% (~0.340 μM) and 56% (~0.280 μM), respectively, versus 37% (~0.180 μM) in the control condition (Figure 4B,C). The increase in extracellular ATP induced by DTT and FGFs was significantly reduced after treatment with CBX and ENX, reaching control levels (Figure 4B,C). 

Then, the intracellular ATP shows a value of 66%, which slightly decreased to 61% (DTT) and 64% (FGFs) (Figure 4B,C). The presence of CBX and ENX caused a significant increase to 75% (Figure 4B) and 89% (Figure 4C) in the intracellular ATP (leaking block), presenting a direct correlation between extracellular/intracellular ATP. These results suggest that HCs-Cxs mediate ATP release in response to changes in redox potentials and/or growth factors. In turn, CBX/ENX reduces ATP release generating concentration-dependent NTDs, additionally suggesting the functional participation of HCs-Cxs in the closure of the neural tube.

### 2.5. In Silico Analysis: Study and Analysis of Potential Interaction Sites in Connexin 46 Hemichannels

Our results suggest that Cxs participate in the neurulation process by forming functional hemichannels. On the other hand, CBX and ENX are known to block the function of these proteins [64]. We decided to explore the molecular background associated with this mechanism. Surprisingly, it is unknown exactly which domains allow Cxs to interact with pharmacological blockers. The three-dimensional structure of Cx46 is available [68] and was used to elucidate the interactions between HCs-Cx46 and pharmacological blockers through in silico tools, allowing us to predict the possible interaction sites of these blockers on the HCs-Cx46 structure. 

**Figure 5:**
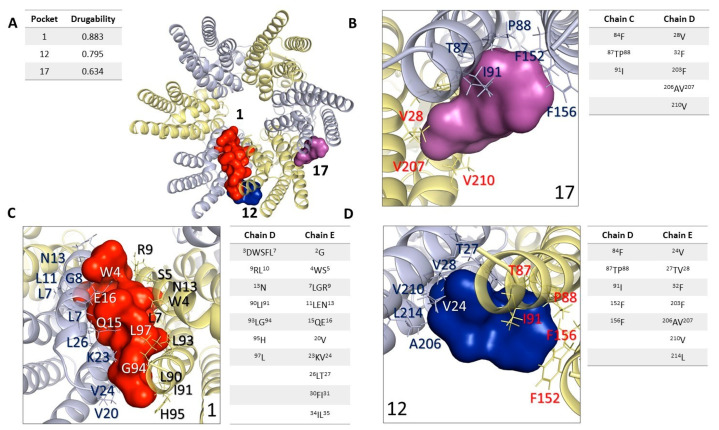
Cavities identified in the hemichannel of connexin 46. Hemichannel is visualized in a 90° rotation (x-axis). (**A**) Structure of HC-Cx46 representing the three best cavities; cavity 1 = 0.795, cavity 12 = 0.883, and cavity 17 = 0.634 calculation carried out with Fpocket. (**B**–**D**) The chain structure of Cx46 represents the three best cavities found by Fpocket. Summary table, detailing drugability score, amino acid residues, chains, and domains.

In the first stage, we identified binding cavities using the structure of HCs-Cx46 and the Fpocket software detecting 3 pockets with a score higher than 0.5 that have been empirically associated with the minimum value of a drug (Figure 5) [69,70], which we call cavity 1, cavity 12 and cavity 17 with drugability values of 0.795, 0.883 and 0.634, respectively (Figure 5A). Cavity 1 is found between two chains (chains A-B) in the N domain. The amino acid residues associated with the cavity in chains A and B are shown in Figure 5B. Cavity 12 presented the best drugability score = 0.883 of the 3 cavities. The residues that form this cavity are shown in Figure 5C, these residues make up the N domain, TM2, and TM3. The residues found in cavities 1 and 12 are part of the N region. The N of HCs-Cx46 folds in the cytoplasmic vestibule, where they form a construction site, and are well positioned to function as a selectivity filter or activation domain that is common to all connexin isoforms [68,71], for which we identified them as “exposed” CBX/ENX binding sites.

Cavity 17 is a pocket by residues shown in Figure 5D. These residues are in the LE1-LE2 domains of the C chain of HC-Cx46, which are located at the coupling interface to form the gap junction [43,68,72].

### 2.6. In Silico Analysis: Molecular Docking Simulations, Connexin 46 Hemichannel Interactions with Carbenoxolone/Enoxolone

**Figure 6:**
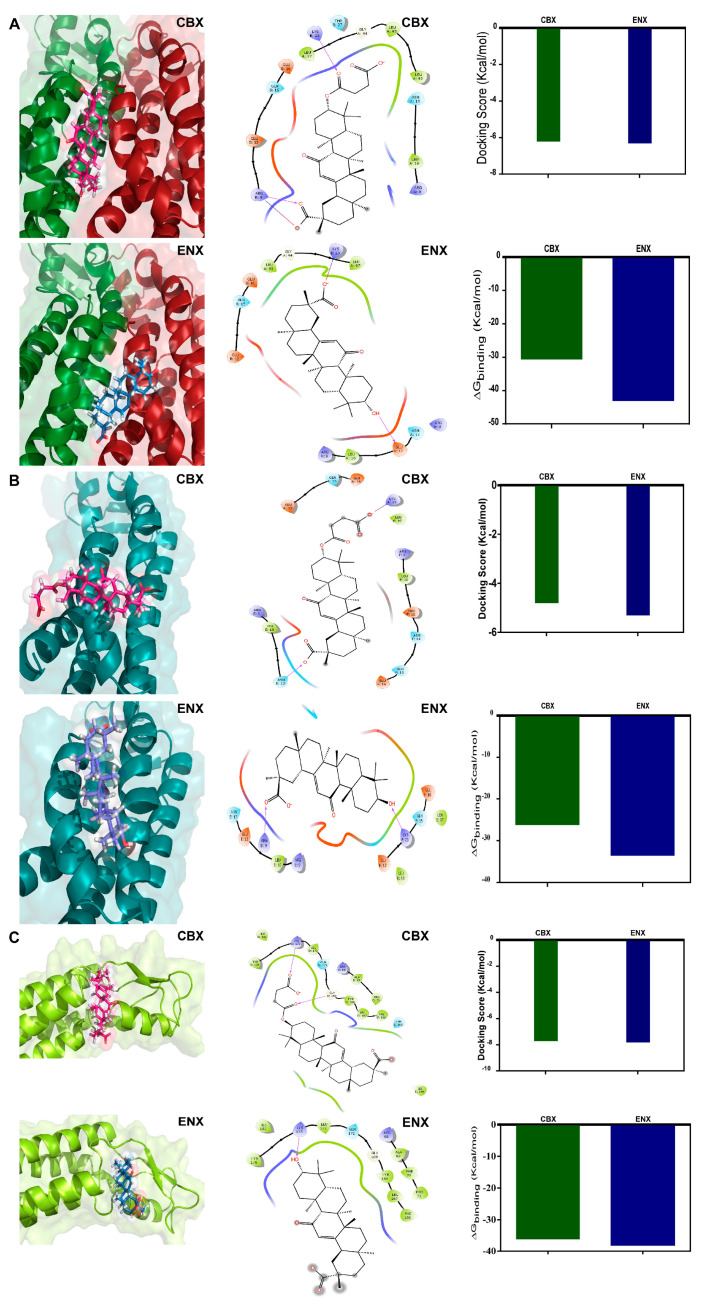
Molecular docking of CBX and ENX in the structure of HCs-Cx46. (**A**–**C**) Representative interaction between HCs-Cx46, CBX (magenta), and ENX (light blue) in the three cavity predicted previously. All the chains are identical, each monomer is represented with a different color to facilitate the identification of the regions between subunits. Comparison of CBX/ENX docking in the NT domains of HCs-Cx46. 2D diagram of the interactions identified for HCs-Cx46. The CBX and ENX binding site residues that interact with the NT of HC-Cx46 are shown schematically and colored according to their physicochemical properties. The arrows indicate the directionality of the hydrogen bond and the degraded line represent a salt bridge. Plots of docking scores and theoretical binding ΔG between CBX/ENX interactions with HCs-Cx46.

Next, we analyzed the ability of the amino acids of each cavity to interact with CBX and ENX, using the AutoDock Vina [73]. Molecular docking simulations on the structure of HC-Cx46 and a focused search on cavities 1, 12, and 17 were performed. The HC-Cx46/CBX complex of cavity 1 (Figure S3A), presented interactions with a binding energy of −6.2 Kcal/mol (Figure 6A) while the HC-Cx46/ENX complex (Figure S3B) show a similar binding energy of −6.3 Kcal/mol (Figure 6A). For the HC-Cx46/CBX and HC-Cx46/ENX complexes of cavity 12 (Figure S3C,D), we determined interactions with a binding energy of −4.8 Kcal/mol and −5.3 Kcal/mol, respectively (Figure 6B). According to these results, we predict that cavities 1 and 12 located in the N region show similarities in molecular docking with CBX and ENX. Both compounds can interact structurally and energetically favorably with Cx46. 

In cavity 17 of the HC-Cx46/CBX complex (Figure S3E), we calculated a binding energy of −7.7 Kcal/mol (Figure 6C) slightly higher than that obtained in the HC-Cx46/ENX complex (Figure S3F) with −7.8 Kcal/mol (Figure 6C).

The analysis for site 1 (corresponding to cavity 1) shows that CBX and ENX bind preferentially to polar amino acid residues that belong to the N domains of two chains of the hemichannel (Figure 6A). At the HC-Cx46/CBX interface, we observed the formation of two H bridges with the amino acids Arg9 (+) and Lys23 (+) and a salt bridge interaction with Arg9 (+) (Figure 6A), these interactions contribute to the stabilization of the complex, reaching a ΔG = −30.61 Kcal/mol (Figure 6A). In the HC-Cx46/ENX interaction of site 1, we show the presence of an H bridge with Glu12 (−) and a salt bridge with Lys23 (+) (Figure 6A). These interactions, stabilize the complex, reaching a ΔG= −43.05.61 kcal/mol (Figure 6A). The calculated binding energy indicates an energetically favorable coupling between CBX/ENX and the HCs-Cx46.

For site 2 (corresponding to cavity 12) (Figure 6B), docking between HC-Cx46 and CBX shows interactions with Asn13 (polar, uncharged) forming an H bridge and a salt bridge with Lys23 (+) (Figure 6B), these interactions reached a ΔG = −26.2 Kcal/mol (Figure 6B). In the HC-Cx46/ENX interface, we found H bridge interactions with Arg9 (+) and Lys23 (+) (Figure 6B), reaching a ΔG of −33.5 Kcal/mol (Figure 6B). We were able to corroborate that sites 1 and 2 form a single pocket (site N), even though the Fpocket program resolved site 2 (pocket 12) as a different pocket than site 1 (pocket 1). Consistently, the N site is formed by Arg9, Leu10, Glu12, Asn13, Gln15, Glu16, Ly23, Leu90, Leu93, and Leu97 of the N-terminal and TM2 domains of HC-Cx46 (Figure 6A,B). The N region has conserved amino acids in the different Cxs isoforms [68,71,74], which would explain why these blockers modulate their activity in their gap conformation junction and hemichannel.

The site 3 (site E) (corresponding to cavity 17) (Figure 6C), we found that CBX and ENX bind to amino acid residues that conform to the LE2 domain. The CBX and ENX molecules interact with the LE2 site composed of hydrophobic and polar amino acids. The HC-Cx46/CBX interaction showed the formation of two H bridges with the amino acids Gly169 and Lys173 (+) (Figure 6C), reaching a ΔG = −36.09 Kcal/mol (Figure 6C). In addition, in the HC-Cx46/ENX interaction, we observed an H bridge with Lys173 (+) (Figure 6C), scoring a ΔG = −38 Kcal/mol (Figure 6C). The extracellular loops of the Cxs described as highly conserved domains part of the coupling mechanism for the formation of functional gap junctions. In particular, LE2 is crucial for the coupling specificity when the channel is formed [65,72], which could be affected by the interactions formed with CBX and ENX. However, these analyzes are not sufficient to discriminate which of the two sites modulates the coupling of CBX and ENX or if they act synergistically. 

Finally, the in silico studies correlate with the functional data obtained in the pharmacological tests, showing that CBX and ENX bind and modulate the function of the Cxs in their conformation of HCs.

## 3. Discussion

### 3.1. Connexins Expression and Function during the Neurulation in Xenopus laevis

Neurulation and the consecutive events required for neural tube formation have been extensively described [1,15,75]. However, what genetic or environmental elements induce failures during these processes causing neural tube defects (NTDs) remain incomplete. It has been described that at least 300 genes participate in the formation of the neural tube [8], despite this, their relationship with neurulation mechanisms and the formation of neural tubes, and the appearance of NTDs have not been detailed.

As mentioned, Cxs HCs can release ATP, Glu, and other molecules to the extracellular milieu while Cxs gap junctions allow direct cell-cell transfer of low molecular weight (<1 kDa) metabolites, and ions, including cAMP, inositol triphosphate (IP3), and Ca^2+^ [41,76,77]. These molecules act as autocrine/paracrine messengers [36,37,39,41,49,51,67,78] regulating physiological and pathophysiological events in neurons, astrocytes, tanycytes, neural precursors (NPs), embryonic stem cells and neural crest cells [35,38,39,42,44,46,48,79,80,81,82]. 

Cxs change their expression dynamically during CNS development in vertebrates [38,44,45,46,47,48]. Then, until now, there are no studies that demonstrate the presence and role of Cxs during neurulation. Here, we provide results demonstrating for the first time the presence of Cx43, Cx46, Cx45, Cx32, Cx26, Cx31, Cx25 transcripts and cx46 protein in different neurulation stages of Xenopus laevis. We clearly show a Cx transcript pattern in which Cx46 was the most represented (100 times more) during neural tube closure (Figure 1) followed by Cx32 (30 times) (Figure 1). Previous studies have shown that Cx subunits change throughout development because of changes necessary for tissue maturation, i.e., the transition from a stem cell to a differentiated state [79]. Embryonic stem cells (ESC) have been shown to express around 18 Cxs, including Cx46, Cx43, Cx32, and Cx26 [79], data that support the pattern of gene expression observed in our results (Figure 1). Bannerman et al. showed that Cx46 and Cx43 form gap channels in early neural crest cell (NCC) migration, evidence suggesting a crucial role of Cxs in signaling during embryogenesis [44].

The transcript expression profile of Cx46 vs. Cx43 (Figure 1B) in neurulation is a relevant finding. A reciprocal relationship between Cx46 and Cx43 has been postulated in the differentiation of glioblastoma cancer stem cells, in which Cx46 is expressed predominantly in undifferentiated glioblastoma cancer stem cells and Cx43 in non-cancer stem cells, suggesting that the Cxs expression specificity in processes such as differentiation are crucial [83]. Our results in Xenopus laevis are consistent with these studies, in which we found that Cx46 is expressed during neurulation when neural cells begin their differentiation process and Cx43 in the adult differentiated brain cells (Figure 1A). Future experiments will be necessary to evaluate the expression and specific location of these proteins (Cx46, Cx43, Cx45, and Cx32, among others) and elucidate the presence, functionality, and composition of HCs and/or gap junction formation during neural tube closure. CBX/ENX affect both HC-Cx and GJs [64] whereas our experiments of extracellular LY incorporation and ATP release evaluate only HC-Cx conformation. GJ functionality has not been tested in this study and we cannot discard its participation especially if a Cx able to form GJ is present as Cx46 is. Future LY intercellular permeability experiments and the use of mutant Cxs unable to form GJ should be performed to evaluate the presence of GJIC conformation and its eventual participation.

Neither the participation of Cxs in the neural tube closure nor their association with the appearance of NTDs has been reported. Here, we demonstrate in vivo that Cxs play a crucial role not only during the neurulation process but also in the development of the anteroposterior axis of Xenopus laevis embryos. Exposure to CBX and ENX, widely Cxs blockers, induce a concentration-dependent effect, affecting the closure of the neural tube, changing the morphology of embryos in later stages of development (stage 45), and causing NTDs. We evidenced a delay in neural tube formation at intermediate concentrations of 30 μM CBX (Figure 2) and 50 μM ENX (Figure S2). The subsequent development of the embryos presented defective phenotypes, with their curved anteroposterior axis, evidencing an alteration in the formation of their spinal cord (30 μM CBX) (Figure 2) or simply, the complete inhibition of growth (100 μM CBX/ENX) and subsequent degeneration and death. Although these results were clear, they must be interpreted with caution because these blockers inhibit the majority of Cxs [64], but currently, there are no specific blockers for particular Cxs, and future experiments using i.e., molecular knockdown with molecular approaches, i.e., morpholine, should be performed. 

Our results agree with those described by other investigations, which have shown that exposure to pharmacological Cxs blockers generates alterations in events such as proliferation, migration, and differentiation in various cell types, especially in neural crest cells [44], neuroblasts [84], neural precursors [47], and embryonic stem cells [79].

### 3.2. FGF2 and Intracellular Redox Status Regulate the Opening of HCs-Cxs during the Neurulation Process in Xenopus laevis

HCs-Cxs participate in autocrine and paracrine signaling releasing ATP, Glu, and others [36,37,39,49,51,67]. Here, we demonstrate that HCs-Cxs, probably participate in the release of ATP during this process. The opening of HCs-Cxs can be activated by (i) FGFs [52,53,54,55] and/or (ii) changes in the intracellular redox potential, which depends on the cysteines located in the CT domain of the Cxs [49,51,57,59]. The participation of HCs-Cxs in neurodevelopmental events and neurogenesis [38,45,48] suggests the presence of modulation mechanisms of HCs in stages such as neurulation. Cxs blockade (ENX/CBX) affected the permeability and ATP release induced by changes in intracellular redox status (induced by DTT) and FGF2 in vivo and in vitro, suggesting the participation of these two mechanisms in the regulation of HCs-Cxs during neurulation.

The family of fibroblast growth factors (FGFs) participates in crucial cell responses during development, such as morphogenesis, migration, proliferation, induction, and differentiation [4,85,86]. Additionally, FGF2 caused a transient opening of HCs-Cxs, modulating the release of molecules such as ATP. The modulation of the incorporation of Lucifer Yellow by HCs-Cxs during the neurulation by FGF2 and CBX/ENX shows that FGF participates in the opening mechanism of HCs-Cxs (Figure 3 and Figure 4). The increased permeability of the cell membrane through HCs-Cxs caused by FGF2 could be due to an increase in HCs on the cell surface [52,55,56] associated with changes in the phosphorylation state in Ser/Thr/Tyr residues as shown previously [57]. Cx46, similar to cx43, cx50, and other connexins, are phosphorylated in several sites regulating their functions [87,88]. In our experiments, we used FGF, which is the signaling pathway that is active during neurulation [4]. FGF can induce Cnxs phosphorylation and modulate its activity [89,90] allowing LY incorporation [61] and ATP release [41,54]. However, the possible participation of other proteins such as pannexins (Pnxs), cannot be discarded with our current methodology. The preliminary results of our laboratory have not found the presence of transcripts of pannexins 1, 2, or 3 using specific primers. However, further studies are required to confirm the role of Pnxs in neurulation [36,53,91,92]. The effect of FGF2 in neurulating embryos caused a ~3.5-fold increase in LY uptake vs. control embryos and it was sensitive to CBX and ENX (Figure 3). Concordantly, FGF2 induces 2-fold more ATP release versus control embryos, an increase that was reversed by the presence of CBX/ENX to concentrations without treatments (Figure 4), suggesting a complementary release mechanism in addition to HCs-Cxs. In this regard, the participation of P2X3,4 and P2Y1,4,8,11 receptors has been demonstrated during the neurulation [25,28,29,30], which is positive signaling feedback could stimulate the opening of HCs of Pnxs (Pnx1) and/or Cxs [93].

DTT decreases the intracellular redox potential, increasing the functional status of Cxs [57,58,59], which in our case would modulate HCs-Cxs promoting the release of ATP during the neurulation process. This statement is based on: (i) DTT increased LY uptake by 5 times in neurulating embryos, (ii) the increase in LY permeability was inhibited by CBX/ENX (Figure 3), and (iii) DTT doubled the release of ATP and t was inhibited by CBX/ENX (Figure 4). Cx43, Cx46, and Cx32 have cysteines located in the CT and LE1/LE2 domains, which can be reduced or oxidized depending on redox conditions [57,58] during neurulation. Then, the ATP release would participate in closing the neural tube, and its blockade would induce NTDs. We postulate two mechanisms that modulate the opening of HCs-Cxs during neurulation: (i) redox potentials that induce changes in disulfide bridge interactions, and s-nitrosylation, among others [58], causing conformational changes, affecting the open/closed state of HCs-Cxs; (ii) FGF2 induces the controlled opening of HCs-Cxs, allowing the release of ATP, attributable changes to the state of phosphorylation/dephosphorylation of the sensitive sites (Ser/Thr/Tyr) of the N and CT regions of Cxs [51,56,57].

HCs-mediated ATP release could activate P2X3-4, P2Y1,8 receptors [25,28,29,30], triggering intracellular signaling [20,23]. This may involve intracellular calcium [Ca^2+^]i, which regulates cell proliferation, migration, differentiation, and cytoskeletal remodeling during embryonic development and neural tube formation [27,94]. Eventually, the alteration of ATP signaling (HCs-Cxs) can lead to NTDs. Additional neurotransmitters, such as Glu, participate in [Ca^2+^]i mobilization and neural tube formation. The inhibition of the NMDAR [21] and glutaminase 1 (GLS1), (glutaminolysis, GLN → GLU) [22], generates alterations in [Ca^2+^]i and neural plate cell migration resulting in NTDs. This suggests that Glu and ATP could act as complementary interplay signaling during neurulation and neural tube formation helping the comprehensive understanding of neural tube defects etiology [21,22,27,29,30,94].

### 3.3. Limitations and Caveats 

The use of non-specific drugs in our studies is an important limitation to be considered. CBX and ENX act on several HCs-Cxs and some other proteins such as Pnx channels and voltage-gated Ca^2+^ channels [58,92]. Similarly, DTT is a broad reducing agent, that reduces exposed cysteines found in different kinds of proteins. Therefore, other proteins affected by the agents may contribute to the changes reported in this study. However, our study opens the research of connexins and related proteins in neurulation. Thus, the contribution of other connexins, such as Cx32 and Cx43, and Pnx to neurulation must be investigated. Unfortunately, specific pharmacological agents that modulate the activity of connexins are limited and we expect that our in silico work contributes to the development of new specific pharmacological tools. In addition, further studies that included the use of morpholines against connexins will be instrumental in confirming the precise role of connexins in neurulation. These strategies will be important for future research to confirm our findings, which add this new signaling to early neural development. 

Importantly, our study reports the expression of connexins during neurulation. However, our study does not address the lack of correspondence between RNA and protein levels of connexins in this experimental in vivo model. This complex issue is beyond the scope of our study and requires further investigations of the complex regulations that governs gene expression in Xenopus neurodevelopment, including transcriptional mRNA processing, maturation, localization, mRNA degradation, mRNA degradation, and translation [95,96] and protein degradation [97,98]. In this vein, Kindt et al., 2017 and Chau et al., 2018 [99,100], showed that the functional annotation clustering of E8.5 mouse embryos (when Neurulation begins) informs that neuroepithelium enriched genes include genes encoding ribosomes/translation, nucleolus/nucleoplasm, ribosome biogenesis, RNA processing, and mitochondrion. Moreover, the functional annotation clustering of E10.5 neuroepithelium-enriched genes shows an overrepresentation of genes for neuron differentiation, cell adhesion, cell migration, cytoskeleton, and transcription regulation [99,100], which highlights the complex gene regulation that occurs during neurodevelopment.

Cx46 is a membrane protein associated with a paracrine (hemichannel) and/or juxtacrine (gap junction) signaling function. It has been shown that protein abundance regulation mirrors specific biological roles, such as, regulatory proteins may have to be produced and degraded very rapidly to react to a stimulus, whereas structural or housekeeping proteins would be much longer-lived [101]. 

With these antecedents, we believe that Cx-mediated signaling is incipient during neurulation. This, and other cellular communication during this period, are highly dynamic processes as a consequence of the accelerated embryo growth (area/mass) and movements may be restricted to a specific region, such as neural tissue, and may be affected by a dilution effect [102], whereas the half-lives of the proteins depend on proliferation state, which should be verified with future experiments. 

Finally, and supporting the comments above, the mRNA levels of cx46 are highly dynamic, varying during the analyzed stages, during Neurulation which may be related to an incipient signaling, different to a sub 1, for example, which corresponds to a stable mRNA signal and it is related to a more basic cellular maintenance transcriptional functions [103].

### 3.4. In Silico Predictions: Binding Sites and Interactions of Widely Used Blockers to Modulate Cxs Activity

Until now the mechanism of action of CBX and ENX on Cxs has not been fully elucidated, in this study, we gather evidence about the interaction of these blockers with Cxs.

The identification and characterization of functional sites, such as ligand binding sites or catalytic sites of a protein structure, are very useful to understand its mechanisms of action [70]. In this study, we combined pocked detection [69,70] and docking simulation to evaluate the possible sites that allow us to predict a theoretical mode of binding blockers widely used to modulate Cxs function on the structure of Cx46. Two candidate sites located in the N and LE2 domains of Cx46 were predicted for CBX and ENX interaction.

The N site region (Figure 5 and Figure 6) is highly conserved among the different Cxs isoforms [68,74] and participates in the pore-forming region, a determining site for the modulation of opening/closing of these channels [57,71]. Although the physiology associated with the modifications in this region has been characterized [71,74], the structural mechanism of small molecules capable of modulating their function such as CBX and ENX is not fully known. Our results suggest that CBX binds more favorably to this site compared to ENX (Figure 6). The N site contains a positively charged amino acid (Lys or Arg) at position 22 (Cxs β) or 23 (Cxs α) that plays an important role in the biosynthesis of Cxs [71] and its interaction with CBX and ENX would affect critical interactions for the formation of HCs, as well as interactions involved in maintaining the open/closed state of these channels. For its part, the LE2 site is located near residues 179–195 crucial in the formation and specificity of gap junctions for Cx46. Cx46 has been shown to be heterotopically compatible with Cx26, Cx30, Cx32, Cx50, and Cx43 [43,65,72]. Interestingly, CBX also generates stronger interactions than ENX at this site. Thus, our predictions correlate with the pharmacological tests where it was shown that CBX generates greater phenotypic defects, altering the later development of the embryos, evidencing an alteration in the formation of their spinal cord (30 μM CBX) or simply, death (100 μM CBX) (Figure 2). From these results, it can be suggested that both N and LE2-located sites are essential for the modulation of the Cxs.

Taken together, we demonstrate that HCs-Cxs participate in the release of ATP during neurulation. We also show that the opening of HCs-Cxs is modulated by signaling pathways as activated by FGF2 and by changes in (ii) intracellular redox potentials. Remarkably we found that the pharmacological blockade of HCs-Cxs induces the generation of NTDs, which opens a new avenue to understanding these types of neurodevelopmental alterations.

## 4. Materials and Methods

### 4.1. Ethical Statement and Experimental Animals

The care and handling of the *Xenopus* model were approved by the National Research and Development Agency (ANID) and by the ethics, Care and Use of Animals committee of the University of Concepción (Fondecyt 11160562). The animals were treated following the ethical protocols established by the United States National Institute of Health (NIH) and the amphibian euthanasia protocol was based on that described by [104].

### 4.2. Obtaining Xenopus laevis Embryos

*Xenopus laevis* oocytes were collected using the in vitro fertilization technique. Ovulation of the female frog was previously induced by subcutaneous injection of the hormone Human Chorionic Gonadotropin (hCG). 48 h before fertilization (pre-prime), the female was injected with 50 units of hCG. 24 h later the female was injected again with 250–300 units of hCG (prime). Embryos were maintained in Marc’s modified Ringer’s solution (10% MMR, pH 7.4), containing 1 M NaCl, 20 mM KCl, 10 mM MgSO_4_, 20 mM CaCl_2_, and 50 mM HEPES. The male gonads were obtained through abdominal dissection. The testes were stored in 1× MMR medium supplemented with 20% FBS. 12 h after the second injection of hCG, the oocytes were collected in 1× MMR solution, later they were fertilized with *X. laevis* testis extract and incubated for 1 h at room temperature in 10% MMR solution. Afterward, the medium was replaced by a 2% cysteine solution in 10% MMR, pH 7.8–7.9 for 5 min, to dissolve the gelatinous layer “degelatinization” [22]. Subsequently, the embryos were washed and incubated in 10% MMR at room temperature and finally harvested at different neurulation stages: 12.5 (early neurulation), 14 (middle neurulation), and stage 20 (late neurulation); and stage 40–45 (tadpole) [2,105]. We observed the expression of Cxs in stages 12.5–20 and the analysis of the closure of the neural tube and morphological effects in stages 40–45. Stage 10 (gastrulation) was used as a negative control for Cxs expression and the adult brain as a positive control to validate Cxs expression in molecular biology assays.

### 4.3. Conventional RT-PCR and Real-Time RT-qPCR

#### Total RNA Extraction

For the total RNA extraction of the embryonic stages of *X. laevis*: Stage 10, stage 12.5, stage 14, stage 20, and adult brain tissue, the EZNA^®^ HP Total RNA kit was used, homogenizing the samples in 700 μL of the buffer of GTC lysis, this volume was implemented according to the manufacturer’s instructions per amount of tissue (30 mg) (15 embryos). The samples were subjected to centrifugation and washed with 500 μL of 70% *v*/*v* ethanol according to the Kit instructions. Finally, the total RNA was resuspended in 30 μL of RNase-free water and quantified by measuring its absorbance at 260 nm and its purity according to the 260/280 ratio in the NanoQuant infinite 200 PRO equipment, Tekan. RNA was stored at −80 °C for later use.

### 4.4. Reverse Transcription (RT) of Total RNA

DNA synthesis was performed using the reverse transcriptase enzyme M-MuLV Enzyme Mix (ProtoScript^®^ II First Strand cDNA Synthesis Kit, New England BioLabs, Ipswich, MA, USA), adding 1 μg of each total RNA sample. For a final volume of 20 μL, the RNA was incubated with 0.5 μg of oligo-dT, denatured at 70 °C for 5 min, and placed on ice for 2 min. Subsequently, 400 U of the reverse transcriptase enzyme M-MuLV Enzyme Mix was added and incubated for 1 h at 42 °C and finally at 70 °C for 5 min. The negative controls for the amplification of the samples were treated with the same transcription protocol, but without adding oligo-dT or reverse transcriptase enzyme to the mixture.

### 4.5. cDNA Amplification by PCR

The cDNA amplification was conducted in a Bio-Rad thermocycler (Icycler, Temecula, CA, USA) implemented with the PCR kit (New England BioLabs, MA, USA). In a mixture containing 1X ThermoPol or Standard Taq Reaction Buffer, a mixture of dNTPs (0.2 mM each) and the following set of specific primers (500 nM each) (Table S1), Taq DNA polymerase 0.625 U (New England BioLabs) and 1 μL of the reverse transcription product, in a final volume of 25 μL. The incubation program consisted of 95 °C for 5 min, followed by 35 cycles of 95 °C for 30 s, 57 °C for 30 s, 72 °C for 30–40 s, and a final extension at 72 °C for 5 min. The synthesis of all cDNAs was tested using specific primers for the Sub-1 transcription gene [103] to normalize the experimental results [106]. The primers were designed using the NCBI Primer-Blast software (https://www.ncbi.nlm.nih.gov/tools/primer-blast/ accessed on 1 January 1989), considering their Tm, the percentage of GC, the formation of dimers and structures. 

### 4.6. Agarose Gel Electrophoresis

DNA fragments were separated on 1% agarose gel. Gels were prepared in TAE electrophoresis buffer (Tris-Acetic Acid EDTA; 40 mM Tris-HCl, 30 mM acetic acid, and 1 mM EDTA; pH 7.6 and 0.5 μg/mL ethidium bromide (APEX, Houston, TX, USA). The molecular weight marker of 100 bp DNA ladder (GeneRuler, Thermo Scientific, Waltham, MA, USA) was used. To visualize the different DNA bands in the agarose gel, transilluminator equipment was used.

### 4.7. Quantitative Real-Time RT-PCR (qRT-PCR)

The real-time RT-qPCR reaction was prepared with the Brilliant II SYBR Green qPCR Master Mix kit (Agilent Technologies, Santa Clara, CA, USA) in a final volume of 20 μL, containing 2 μL of cDNA and 500 nM of each splitter set (Table S1). Each reaction mixture was incubated at 95 °C for 5 min, followed by 40 cycles at 95 °C for 30 s, 55 °C for 30 s, 72 °C for 30 s, and a final extension at 72 °C for 5 min, in an Mx3000P thermocycler (Agilent Technologies, Inc., Santa Clara, CA, USA). The relative expression of Cxs was calculated by the ΔCT method using sub-1 as the reference gene [103]. Subsequently, the ΔΔCt was determined by normalizing the conditions to stage 10.

### 4.8. Pharmacological Modulation of the Neurulation Process

Stage 12.5 embryos were incubated with CBX (Tocris, Bristol, UK) at 3, 5, 10, 20, 30, 50, 100, 200, and 300 μM and ENX at 3, 5, 10, 20, 30, 40, 50, 60, 70, 80, 100, 200, and 300 μM and with MMR saline as a control. After 7 h of incubation, the embryos (stages 19–20) were washed three times with 10% MMR and kept in this solution until the tadpole stage (stage 40–45) [22]. The analysis and taking of images were conducted in a NIKON model SMZ25 stereo microscope, coupled to a brightfield Amscope camera, 1× objective (embryos stage 20) and 0.63× objective (tadpole embryos) in which the morphological effects on the neurulation process. The quantification of IC_50_ for CBX and ENX was conducted in the ImageJ software, in which the percentage of altered phenotype (incorrect closure of the neural tube, stage 19–20) and alterations in the anteroposterior zone (stage 40–45) were analyzed. The IC_50_ was used in the dye uptake assay (LY) and ATP quantification.

### 4.9. Lucifer Yellow Uptake and Fluorescence Detection In Vivo

LY is not capable of permeabilizing the cell membrane except through functional hemichannels, or gap junctions given its physicochemical properties and molecular weight (442.3 Da) [40]. The functionality of the HCs was evaluated through the uptake of LY in embryos at stage 12.5, both under physiological conditions (FGF2) [55], and redox (DTT) [59]. Embryos were incubated for 7 h at 25 °C under different conditions with a final concentration of 0.2% LY, 20 ng/mL FGF2 (Sigma Aldrich; Cat # SRP4037, St. Louis, MO, USA), 1 mM DTT, 30 μM CBX and 50 μM ENX. Compounds were added in 10% MMR for a final volume of 500 µL. For fluorescence detection, the embryos were washed three times with 10% MMR, pH 7.4, to favor the closure of the hemichannels. The analysis and taking of images were performed in a NIKON (Japan) model SMZ25 stereo microscope, coupled to a Fluo Flir camera, and they were recorded every 60 s with a 1× objective, with 8× magnification (stage 20). The area of the neural plate (NP) was defined as a region of interest (ROI), an area of 0.15 mm^2^ was defined, and LY positivity was quantified with the ImageJ program, in which the average change in fluorescence intensity was measured of LY-positive cells per area.

### 4.10. Quantification of Extracellular and Intracellular ATP

Embryos were placed in 48-well plates in 10% MMR, treated with a final concentration of 20 ng/mL FGF2 [55], 1 mM DTT [59], CBX 30 µM, 50 µM ENX and 100 µM gramicidin A. The final volume in each well was 300 µL. All experiments started at stage 12.5, and ended at stage 20 (7 h), the concentration of extracellular ATP contained in 100 μL of supernatant and the concentration of intracellular ATP (mechanical lysate of 3 embryos) were measured through the assay luciferin/luciferase bioluminescence assay (ATP Bioluminescence Assay kit; A22066 Thermo Fisher Scientific, MA, USA). Gramicidin A is a molecule that forms pores in the membrane, allowing ATP to leak into the extracellular medium, which is why it was used as a positive control. The amount of ATP in each sample was calculated from a standard curve, whose concentration range includes 0.01; 0.1;0.5, 1, and 10 µM. The absorbance was measured at 560 nm for 2 min at 28 °C.

### 4.11. Western Blot Assay

#### Total Protein Extract

*Xenopus laevis* embryos in neurulation stages were used in this assay. Total protein extracts were manually homogenized (20 Gauge Needle/Syringe) using protease inhibitor (ROCHE, Switzerland). The concentration of total lysed protein was determined using a Micro BSA kit (Thermofisher, Waltham, MA, USA) and NOVOstar equipment (BMG Labtech, Ortenberg, Germany).

### 4.12. Polyacrylamide Gel Electrophoresis and Electrotransfer

The denaturing gel of acrylamide 12% (SDS-PAGE) was used to separate the protein of interest. Samples were incubated at 95°C for 5 min with loading buffer (62.5 mM Tris-HCl pH 6.8, 2% SDS, 10% glycerol, 0.01% bromophenol blue) and 100 mM DTT. 125 µg of total protein were loaded on gel next to the protein standard (Spectra Multicolor, Broad Range Protein Ladder, Thermo Scientific, MA, USA), and run at 100 V in run solution (25 mM Tris, 250 mM glycine, and 0.1% SDS). Proteins were transferred to Immobilon-P membrane (0.45 mm pore, Merck Millipore, Tullagreen, Carrigtwohill, Irland) with transfer solution (25 mM Tris, 192 mM glycine, 20% methanol) at 250 mA during 2 h.

### 4.13. Protein Immunodetection

Several washes with TBS-Tween (150 mM NaCl, 10 mM Tris, 0.05% Tween20) were performed. The membrane was blocked with 5% milk in TBS-Tween for 1 h. Overnight incubation at 4 °C using primary antibodies was carried out. The primary antibody was CT-CX46, with an antigen sequence: CRLPSRNSRHSSNRS (1:250, GeneScript, Piscataway, NJ, USA). The secondary antibody incubation was performed for 1 h using anti-rabbit peroxidase-conjugated antibodies (1:1000; Jackson ImmunoResearch Laboratories, Inc., West Grove, PA, USA). The membrane was washed with TBS-Tween for 10 min three times. Finally, the membrane was revealed with a chemiluminescent solution (Western Lightening Plus-ECL, Perkin Elmer, Waltham, MA, USA) in the chemiluminescent and fluorescent equipment (Odyssey FC, Li-COR, Lincoln, NE, USA). For dot blot experiments, 25 μg of total protein extract was used and a similar detention procedure was used. 

Densitometric quantification was achieved using Image J software (NIH). The signal associated with cx46 which included the signal area (ROI) and the intensity was performed using the parametric software’s tool plugin. The measures corresponded to the dynamic range over the background signal. Only a clear and distinguishable peak from the background signal was used. Values below 110 arbitrary units (background) were not considered. 

### 4.14. Bioinformatic Analysis for Hemichannel Interaction Cx46-Carbenoxolone/Enoxolone Selection of Molecules

The structure of Cx46 was extracted from the Protein Data Bank PDB (6MHQ), 3.4 Å resolution [68]. The structures of CBX (CID: 636403; MW: 570.8 g/mol) and ENX (CID: 10114; MW: 470.7 g/mol) were obtained from the PubChem and prepared using the LigPrep program (Schrödinger, LLC, New York, NY, USA, 2016). Charges, protonation states, and rotatable bonds were assigned in the AutoDock Tools version 1.5.6 program [73], before molecular docking simulations.

### 4.15. Preparation of Cx46

The structure of Cx46 was modified in PyMOL version 1.7 [107] to create the hemichannel (HC-Cx46) conformation. The pKa values of the ionizable groups at physiological pH 7 ± 0.2 were predicted in PROPKA [108]. The energy minimization of the structure was performed in MacroModel (Schrödinger, LLC, New York, NY, USA, 2016) in a conjugate Polak-Ribière energy gradient up to a convergence of 0.05 kJ/mol, using the OPLS_3 force field in aqueous solvent.

### 4.16. Prediction of Binding Sites in Cx46

The identification of binding sites of small molecules in the HCs-Cx46 was carried out in the Fpocket program, detecting the protein “interaction pockets” in the modified structure 6MHQ (HCs-Cx46), the detection was carried out by executing the software according to recommended protocol workflow [69,70].

### 4.17. Protein-Ligand Docking

The HCs-Cx46-CBX/ENX interaction analysis was performed in the AutoDock Vina v1.2.3 program [73]. The interaction parameters were made keeping the protein rigid and the ligands as flexible molecules. In addition to this, a screening box or “Grid Box” with specific dimensions centered on the “interaction pocket” was generated using the AutoDock tools program. The generated complexes were classified according to the affinity constants predicted in kcal/mol. The analysis of the HCs-Cx46-CBX/ENX interface and the energetic parameters were performed in the Maestro program (Schrödinger, LLC, New York, NY, USA, 2016). Additionally, the theoretical Δgbind was calculated through the MM-GBSA energy calculation using the Prime program (Schrödinger, LLC, New York, NY, USA, 2016). All images were created in PyMOL v1.7 [107].

### 4.18. Statistical Analysis

Results were expressed as mean ± standard deviation and significant differences were determined using one-way ANOVA (non-parametric) with Dunnett and Bonferroní multiple comparison tests. Values of *p* < 0.05 were considered statistically significant using GraphPad Prism 7.0 software. All experiments were performed at least four times in duplicate for each trial.

## Data Availability

The data that support the findings of this study are available from the corresponding author, PAC, upon reasonable request, and with approval from the ethical committee of Universidad de Concepcion.

## Supplementary Materials

The supporting information can be downloaded at: https://www.mdpi.com/article/10.3390/ijms24032159/s1.
