# Supplementary material for: Understanding the Role of ATP Release through Connexins Hemichannels during Neurulation
Source: Int J Mol Sci. 2023 Jan 21;24(3):2159. doi: 10.3390/ijms24032159 (PMC9916920; doi:10.3390/ijms24032159)
Supplement: Supplementary file 1 [file ijms-24-02159-s001.zip › ijms-2053205-supplementary.pdf]

Supplementary Materials

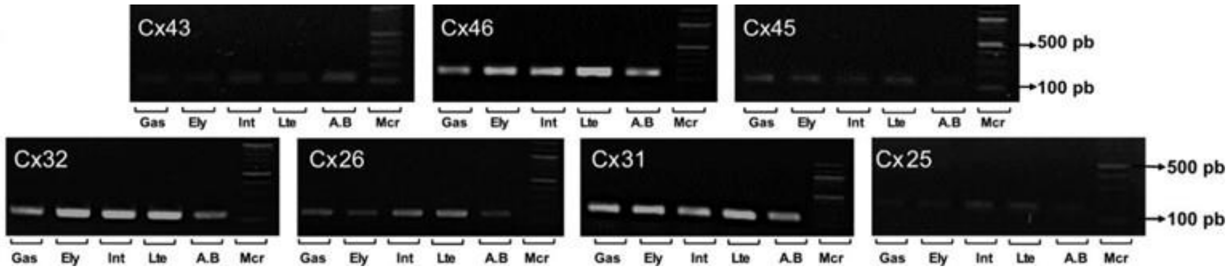

Figure S1. RT-PCR for Cx43, Cx46, Cx45, Cx32, Cx26, Cx31 y Cx25 at different stages of the development of *Xenopus laevis*. Lane 1. Gas (Gastrula stage 10), Lane 2. Ely (early stage 12.5), Lane 3. Int (Intermediate stage 14), Lane 4. Lte (Late stage 19-20), Lane 5. A.B. (Adult Brain) and Lane 6. Mcr (Marker 100 bp).

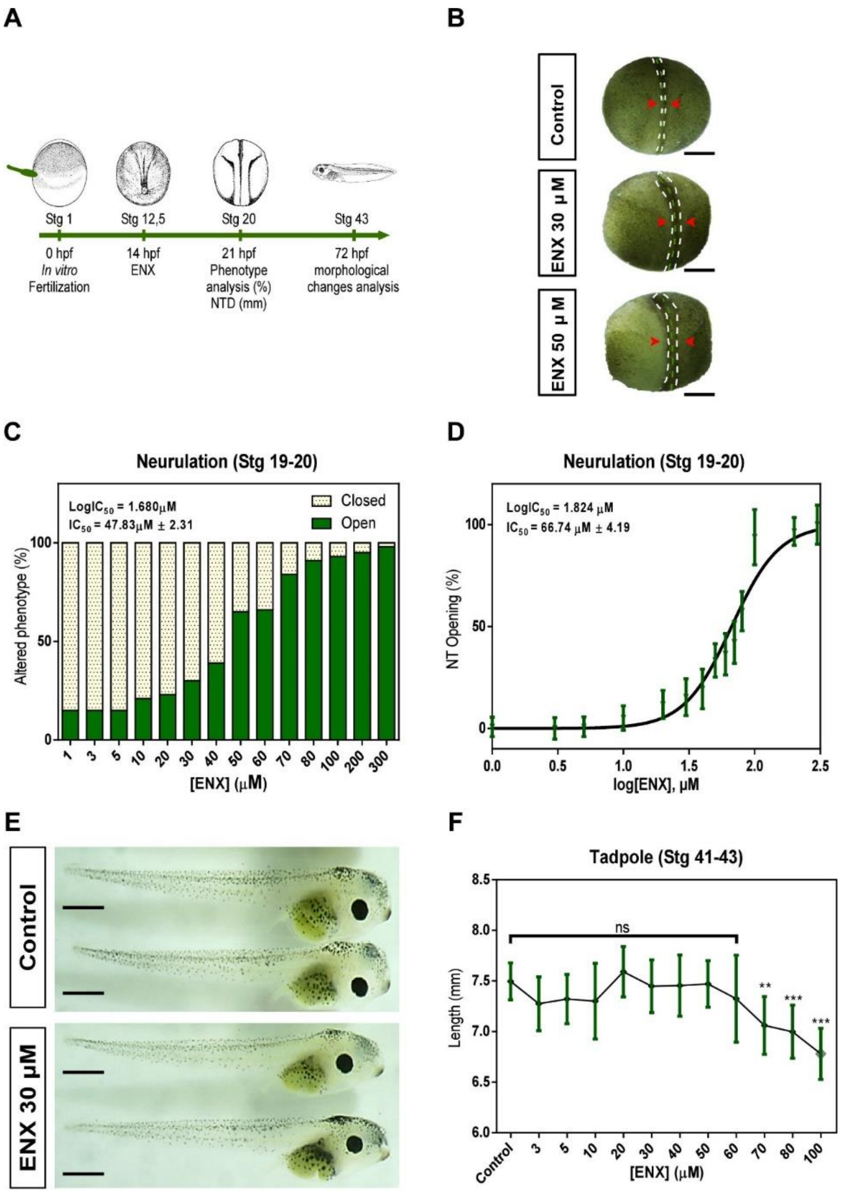

Figure S2. Pharmacological blockade of Cxs with ENX induces NTDs. A. The diagram depicts the stage (Stg) where ENX was initially applied to embryos (Stg 12.5 = 14 hpf). B. Representative photographs of embryos at stg 20 under control conditions (top) or treated with ENX (30 or 50 μM, middle and bottom, respectively). Dashed white lines mark the border between superficial neural and non-neural ectoderm, dashed black lines indicate midline. Red arrow heads indicate the distance of neural folds (closed phenotype= normal; open phenotype= abnormal); scale bar = 500 μm C. The graph shows the percentage of open and closed phenotype of embryos treated in the absence (control= ctl) or presence of ENX (3-300 μM; IC50= 47.83 μM ± 2.31) D. Quantification of open phenotype in embryos at stg 20 treated with ENX (IC50= 67.74 μM ± 4.19). E. Phenotype of tadpoles (stg 41-43) treated with ENX at stg 12.5-20. Scale bar = 1 mm. F. The graph shows the effect of CBX on the length of Xenopus tadpoles. Independent fertilized embryos (N=6) were analyzed for condition (10 experimental replicates). One-way ANOVA, Dunnett's correction, ** = p<0.01; *** = p<0.001; ns= not significant). Error bars represent the standard deviation.

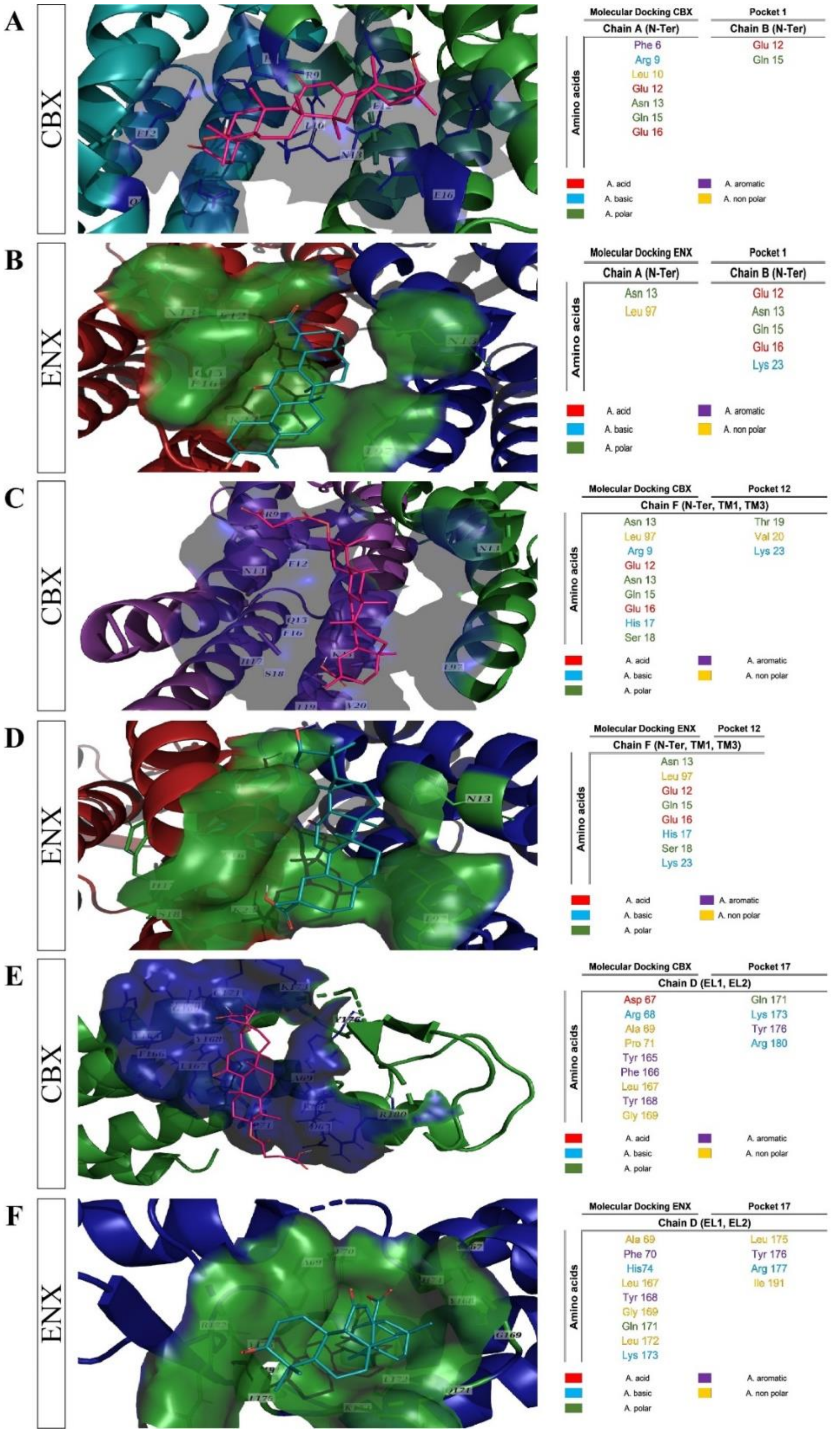

Figure S3. Molecular docking and involved amino acids of HC-Cx46 with carbenoxolone and enoxolone. A-F. Determination of CBX and ENX interaction site in HCs-Cx46, determined AutoDock Vina; summary table of amino acid residues involved in HC-Cx46/CBX (A, C, E) and HC-Cx46/ENX (B, D, F) interaction. Interactions with best binding energy score (-6.2 Kcal/mol (pocket 1); -4.8 Kcal/mol (pocket 12); -7.7 Kcal/mol (pocket 17) CBX) (-6.3 Kcal/mol (pocket 1); -5.3 Kcal/mol (pocket 12); -7.8 Kcal/mol (pocket 17) ENX). In blue (CBX) and green (ENX), regions identified for the docking of pharmacological blockers in HCs-Cx46.

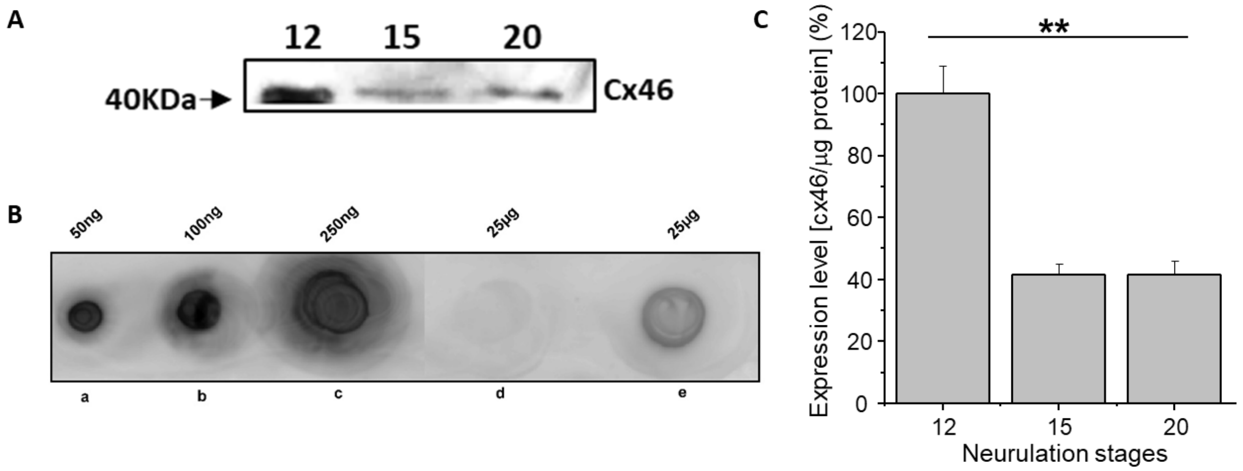

Figure S4. Cx46 is expressed during Neurulation in Xenopus laevis. **A,** Representative western blot showing the expression of cx46 in different stages of Neurulation in Xenopus. Lane 1 corresponds to stage 12; lanes 2 corresponds to stage 15; lane 3 corresponds to stage 20. A molecular weight marker is indicated with an arrow. **B.** Densitometric quantification of cx46 signal normalized by the total amount of protein loaded in each lane. Bars correspond to average ± ES. (** = $p<0.01$; $n=4$). **C.** Dot Blot Assay Anti-Cx46. a-c. Positive control (peptide sequence: CRLPSRNSRHSSNRS). d. Mouse cerebral Cortex. e. *Xenopus laevis* embryos stage 20.

Table S1. Set of primers used for RT-qPCR.

Gene	Sense 5'-3'	Antisense 5'-3'
**Cx43**	CCT GGG GCG ATG AAC AAT CA	ACC CAG AAA CGC ACA TGA GA
**Cx38**	CTGCCAGATGCTGTGAAACAT	GTCCACACAGACTCACCTGC
**Cx46**	TGCCACTGGCTGAGATGTGAC	ACCTTTTCGGCAGCTGATCC
**Cx37**	ATTGCTGGGGGAATACTAAGATCA	TCAGACTGCTCATCTCCCCA
**Cx45**	GGC TGA CGG TGC TCA TCA TA	AGG GGT GCA AAT GCA TCG TA
**Cx59**	ACCTCCACAGTAAGCCACAC	TGGGATGGTACCCACATGGA
**Cx62**	CTCCATTGAGGGGGTCCCTA	TATTAGGGCAAGGGGACCGT
**Cx32**	CAGGAGAGCAGCTAACACATCT	CCGCCACAAGCACCATGATA
**Cx26**	TCAGCGCACAGAGACCAATA	AAATCGGACTGTTCGTCCCC
**Cx31**	TCCGTCCCAAGGTTGTCATT	AACTTTCTCTGCTGCGACCA
**Cx31.1**	GTATCCCATGTCCGCCTCTG	AATACTGAGTCCACTGCGGC
**Cx25**	GCTGCTTTTGAACATTTCATTGTCG	GCTTCGGCTGCAACAACATA
**Cx46.6**	GCTCAGGAACAAAACTGCCC	CTCCTTGCTTTTCGTGAGCG
**Cx30.2**	TTGGGCCCTACAGCTAGTGA	AAGACTTCCCGCATCACACA
**Cx40.1**	TGCACGGGGCAGTATTTTCT	TCTCTCTTTAGGCGCTCGGA
**Sub1**	AGC AGG AGA AAT GAA GCC AGG	CCG ACA TCT GCT CCT TCA GT
